# Arsenic and the Trembles

**Published:** 1842-03

**Authors:** 


### ARSENIC AND THE TREMBLES.

We understand from authority on which we can rely, that Dr. Seaton, a new champion of the old hypothesis that arsenic is the cause of Trembles, has actually succeeded in killing a cow with that poison!

We trust that no one will hereafter doubt its being the cause of that disease. To the incredulous we present the following syllogism, which may help them out: Cattle die of Trembles—a cow died from taking arsenic, *ergo*, the Trembles are produced by arsenic: *quod erat demonstrandum.* D.

